# Comparative Biology of Centrosomal Structures in Eukaryotes

**DOI:** 10.3390/cells7110202

**Published:** 2018-11-08

**Authors:** Ralph Gräf

**Affiliations:** Department of Cell Biology, University of Potsdam, Karl-Liebknecht-Str. 24-25, 14476 Potsdam-Golm, Germany; rgraef@uni-potsdam.de; Tel.: +49-331-9775520

**Keywords:** centrosome, centriole, cilium, basal body, spindle pole body, SPB, nucleus-associated body, NAB, microtubules

## Abstract

The centrosome is not only the largest and most sophisticated protein complex within a eukaryotic cell, in the light of evolution, it is also one of its most ancient organelles. This special issue of “*Cells*” features representatives of three main, structurally divergent centrosome types, i.e., centriole-containing centrosomes, yeast spindle pole bodies (SPBs), and amoebozoan nucleus-associated bodies (NABs). Here, I discuss their evolution and their key-functions in microtubule organization, mitosis, and cytokinesis. Furthermore, I provide a brief history of centrosome research and highlight recently emerged topics, such as the role of centrioles in ciliogenesis, the relationship of centrosomes and centriolar satellites, the integration of centrosomal structures into the nuclear envelope and the involvement of centrosomal components in non-centrosomal microtubule organization.

## 1. Introduction

**Figure 1:**
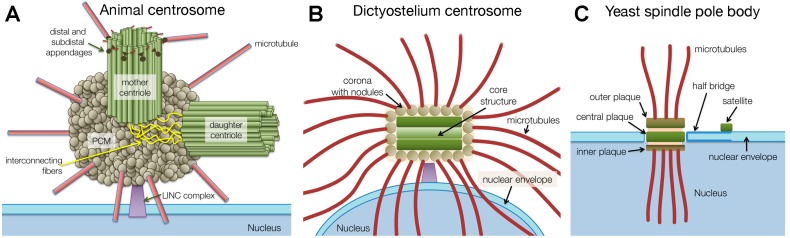
Schematic comparison of centrosomal structures in animals (**A**), *Dictyostelium* (**B**), and budding yeast (**C**). Functionally or topologically related structures are drawn in corresponding colors.

The centrosome is a non-membranous, nucleus-associated organelle that functions as the main microtubule organizing center (MTOC) in many eukaryotes and thus, also as an organizer of the mitotic spindle. With a number of, in some cases, more than 100 different proteins and a size of more than 0.5 µm the centrosome is the largest and most elaborate protein complex in a eukaryotic cell. In animal cells, the centrosome consists of a pair of cylindrical arrangements of short microtubules, called centrioles, which are embedded in a pericentriolar matrix (PCM) serving as a scaffold for microtubule-nucleation complexes. In vegetative cells, the whole structure is linked to the nuclear envelope and nuclear lamina through LINC (linker of the nucleus and cytoskeleton) complexes (see [1] and references therein). Yet, centrioles are absent in many fungi and amoebozoans. Instead their centrosomal structures consist of various plaque-like structures that are also associated with a microtubule-organizing matrix. In fungi, they are often called spindle-pole bodies (SPBs) or, as in amoebozoans, nucleus-associated bodies (NABs) (Figure 1) ([2], Ito and Bettencourt-Dias in this issue of *Cells* [3]). In this review, I will use the term centrosome to subsume all these structures, as evolutionarily related organelles fulfilling common functions should be addressed with a common name. I will use the common abbreviations SPB and NAB when specifically referring to fungal or *Dictyostelium* centrosomes, respectively. While the function as an MTOC and all associated functions related to microtubules are common to all known centrosomal structures in various eukaryotes, the centrosome’s involvement in cell locomotion through cilia and its related role in signaling pathways are restricted to centriole-containing centrosomes.

## 2. Centrosome Research Retrospective

**Figure 2:**
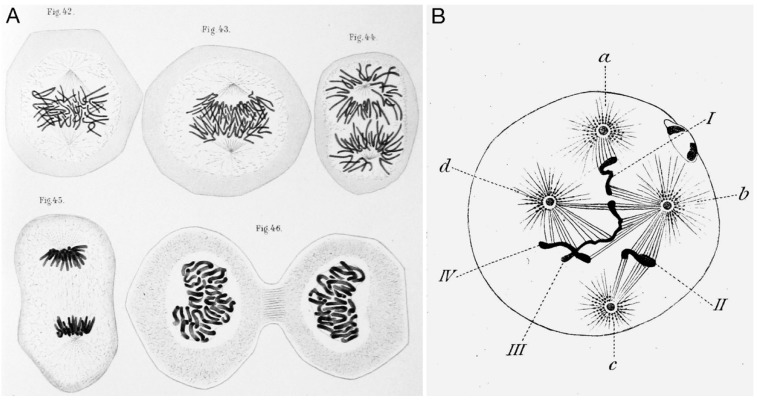
Historic drawings of mitotic figures. (**A**) Salamander peritoneal endothelial cells by Walther Flemming, 1882 [4]. (**B**) Fertilized *Ascaris* egg with multipolar spindles and unequal distribution of chromosomes by Theodor Boveri, 1888 [5]; public domain because of age.

Starting from the first description of an enigmatic organelle involved in the meticulous separation of two sister chromatids into two daughter nuclei in the late nineteenth century, it took more than a century until the centrosome disclosed its secrets. The early work, published around the 1890s is inevitably associated with the names of three famous early cell biologists, the Belgian Edouard van Beneden and the two Germans Theodor Boveri and Walther Flemming. The now common terms “centriole”, “chromatin”, and “mitosis” go back to Walther Flemming, who developed novel techniques to stain tissues derived from salamander gills and fins. He produced numerous, detailed drawings of dividing cells and first postulated that the filamentous structures forming the mitotic spindle are responsible for transport of chromatids and that all nuclei originate from nuclei (“*omnis nucleo ex nucleo*”) (Figure 2A). His main body of work was published in 1882 in his ground-breaking book “Cell substance, nucleus and cell division” [4].

The term “centrosome” was first introduced by his colleague Theodor Boveri (see Müller–Reichert and co-authors in this issue of *Cells* [6]). By observing cell divisions in fertilized nematode eggs he and van Beneden independently found that this self-replicating organelle was the main organizer of cell division [7,8]. Thus, the still valid “once-and-only-once” rule in centrosome duplication goes back to their findings in 1887 [7]. Boveri also realized that centrosomes determine the planes of cell division and that overduplication of centrosomes leading to supernumerary centrosomes results in multipolar spindles. In 1914, Boveri was the first to suggest that the origin of malignant tumors is related to centrosome amplification [9] (Figure 2B). He also was the first to realize that—on a cellular level—chromosomes reflect the hereditary traits postulated by Gregor Mendel three decades earlier. Boveri also first postulated that the first centrosome within a zygote originates from the fertilizing sperm cell, while the unfertilized egg lacks a centrosome [7]. This holds true for many animal species and is discussed in detail in the both the reviews of Avidor–Reiss and Gruss in this issue of *Cells* [10,11]. After these groundbreaking works of the late nineteenth century centrosome research became stuck in its descriptive character and poked along until the late 80s of the twentieth century with a relatively low number of key papers, despite the centrosome’s central importance for cellular function. For a long time, reasons for slow progress in the molecular characterization of centrosomes were: (1) lack of effective centrosome isolation protocols in conjunction with the centrosome’s tight attachment to the nucleus in vegetative cells; (2) the scarcity of centrosomal material (as there is only one centrosome per cell); and (3) the resulting low amounts of mRNAs encoding centrosomal proteins, causing an underrepresentation in cDNA libraries. Thus, it took until 1986 when yeast Cdc31p was the first component of a centrosomal structure to be characterized on the molecular level [12] and until 1988 for its mammalian orthologue centrin [13]. The next milestone was the identification of a new tubulin isoform, γ-tubulin [14], which soon emerged as the key component for our understanding of the centrosome’s role as a microtubule organizer [15]. At that time the origin of centrosomes and their modes of duplication were still mysterious and the discussion whether centrosomes derived from endosymbionts continued and could harbor their own DNA [16], a theory which was finally refuted in the nineties (reviewed by [17]). A few further centrosomal proteins including pericentrin, centriolin, and CP224 were cloned with the aid of autoantibodies from scleroderma patients [18,19] or monoclonal antibodies raised against isolated *Dictyostelium* centrosomes [20]. However, molecular characterization of the majority of centrosomal proteins known to date had to await the completion of the various genome projects and refinement of peptide mass fingerprinting by mass spectrometric methods. 

## 3. Emergence of Centrosomal Model Organisms

With the initiation of genome projects for certain organisms representing the various eukaryotic and metazoan supergroups, model organisms for centrosome research emerged during the nineties of the last century. Centriole-containing centrosomes were mainly studied in the green algae *Chlamydomonas* (see also Wingfield and Lechtreck in this issue of *Cells* [21]) and among animals in mammalian cells, *Drosophila* and *Caenorhabditis elegans* worms. The latter model was particularly useful to study mitosis and spindle assembly in early embryonic development [6]. For acentriolar centrosomes the main models were *Saccharomyces cerevisiae* and *Schizosaccharomyces pombe* as representatives of fungi, and the amoeba *Dictyostelium discoideum*. In the early nineties, ultrastructural data were available mainly for various animal centrosomes, yeasts and *Dictyostelium* amoebae [22]. Due to their lower structural complexity and the expected lower molecular complexity, acentriolar centrosomes from yeast and *Dictyostelium* appeared attractive for molecular and functional analyses, in particular since these organisms provided a much better genetical tractability than animal cells. Moreover, at that time, many researchers interpreted the simplicity of these centrosomes as a sign for an evolutionarily more ancestral structural and molecular composition. Of course, lower complexity promised an easier experimental approach to elucidate the role of the still numerous centrosomal components and their centrosomal functions. Indeed, with regard to the identification of protein components and functions, the yeast spindle pole body was ahead of the game compared to other model organisms, especially in the pre-proteomic era. Availability of the complete genome in conjunction with the establishment of effective procedures for centrosome isolation were the prerequisite for the next milestone, i.e., the disclosure of complete centrosomal protein inventories. Isolation protocols were established for centrosomes from mammalian cells, yeast and *Dictyostelium* [23,24,25,26]. In 1998, budding yeast was the first organism for which an almost complete centrosomal protein inventory was obtained by mass spectrometric peptide mass fingerprinting using isolated SPBs as the protein source [27]. Similar approaches resulted in comparable lists in the order of ~100 centrosomal protein components for centrosomes from mammals, *Drosophila*, *Dictyostelium*, and *C. elegans* [28,29,30,31,32].

## 4. Evolution of Centrosomal Structures

Our current view on centrosomal evolution [33] is not only based on careful analyses of these molecular data in conjunction with structural data [34], it has also been strongly influenced by a revised classification of eukaryotes. According to this, the last eukaryotic common ancestor (LECA) gave rise to five supergroups, the Excavata, SAR (Stramenopile, Alveolata, Rhizaria), Archaeplastida, Amoebozoa, and Opisthokonta together with a few taxonomic side groups of yet undefined relationship [35]. Thus, the idea that simple, acentriolar centrosomes were ancestral to centriole-containing centrosomes was filed away, since it is much more likely that the LECA already possessed centrioles, which were secondarily lost in some amoeboid or sessile organisms after their cilia or flagellae were dispensable for locomotion [3]. According to this theory the primary function of centrioles was the role as a basal body for formation of locomotory cilia and flagellae [21]. In animals, cilia have not only locomotory functions. Many cell types possess primary cilia as sensory organs, e.g., in hedgehog or non-canonical wnt signaling [36]. The loss of centrioles/basal bodies in various phyla and in conjunction with locomotory cilia suggests that the important sensory role of non-motile cilia was acquired only later in evolution. In this issue of *Cells*, Ito and Bettencourt-Dias propose an ancestral PCM core structure that is common to all centrosome types, and that it was this PCM that attracted specific precursor proteins of the duplicating SPB or NAB structures [3]. The latter then replaced centrioles as the core duplicating structures after their loss due to their dispensability in non-motile or amoeboid cells. As highlighted in their paper and also in the contribution of Pitzen et al. in this issue of *Cells* [37], CDK5RAP2 and its orthologues play a key role as PCM scaffolding proteins for γ-tubulin complexes in this context. Yet, the concept that centriole-containing centrosomes are most likely more ancestral than acentriolar centrosomes not at all devalues research on model organisms possessing no centrioles. On the contrary, these organisms are still valuable in comparative centrosome biology as they allow the identification of the shared proteins, which are essential for all centrosomal functions unrelated to cilia/centrioles, i.e., centrosome duplication, nuclear attachment, microtubule organization, and cytokinesis [3]. The first two aspects, centrosome duplication and nuclear attachment, are especially well analyzed in budding yeast and reviewed by Rüthnick and Schiebel in this issue of *Cells* [38]. Due to closed mitosis, in which there is no nuclear envelope breakdown, the newly formed second SPB has to insert into the nuclear envelope in order to allow the formation of a bipolar spindle. The insertion of SPBs into the nuclear envelope shares many similarities with the interphase insertion of new nuclear pore complexes (NPCs) [39,40]. Both processes require a fusion event between the inner and outer nuclear membranes and end up with an inserted large protein complex flanked by a highly-curved membrane. In fact, the conserved component Ndc1 is involved in the SPB insertion network as well as in NPC formation [38]. Similarly in animal cells, NPCs and centrosomes share several proteins such as Gle1, Nup62, Nup133, and Nup188 [41,42,43,44,45] suggesting co-evolution of the two gigantic protein complexes, which most likely were both features of the LECA [46,47]. Insertion of centrosomes into the nuclear envelope is also an important process in a further acentriolar model, i.e., *Dictyostelium* amoebae. Employing mutants with supernumerary centrosomes, Koonce and Tikhonenko report in this issue of *Cells* that only centrosomes engaged at the nuclear envelope are capable of participating in mitotic spindle formation [48]. Furthermore, they show that centrosomes, independently of their nuclear engagement, are capable of driving the formation of cleavage furrows, which are not only important for cytokinesis but ultimately may also lead to the formation of cytoplasts (i.e., nucleus-free cell fragments) containing supernumerary centrosomes. This was first shown in 2003 in *Dictyostelium* cells overexpressing CP224 [49]. Later it was recognized, that the formation of cytoplasts may be one means to control the number of supernumerary centrosomes also in animal tumor cells [50]. 

Moreover, regarding spindle formation, research of the last two decades revealed that the organization of microtubules is much more complex than assumed previously, when the centrosome was considered the sole or at least most important microtubule organizer, especially during mitosis. In 1998, careful analyses using *Xenopus* oocyte extracts revealed that centrosomes are dispensable for spindle formation and that a bipolar spindle can be organized solely by chromatin associated proteins [51]. Here, the small GTPase Ran is rendered into its GTP form through the chromatin-associated GTP/GDP exchange factor Rcc1, which together with Aurora A activates several spindle assembly factors [52,53,54,55]. The latter organize γ-tubulin complexes to nucleate microtubules, which become arranged in a bipolar spindle through the activity of motor proteins including dynein, a bipolar Eg5-like kinesin and a chromokinesin [11]. Further examples for non-centrosomal microtubule organization also in interphase have been especially well-investigated in *Drosophila*. Here the centrosome is not essential for zygotic development and various non-centrosomal MTOCs have been described in different tissues (reviewed by Megraw and co-authors in this issue of *Cells* [56]).

## 5. Recent Developments

After exploration of the protein inventories of the various centrosomal models, the recent decade was marked by the characterization of the protein interaction networks between the numerous centrosomal components. This work began mainly with co-immunoprecipitation assays, tandem-affinity purification approaches and yeast two-hybrid analyses [31,57,58]. Most recently it was driven forward also by proximity-dependent biotin identification (BioID) [59,60]. In this method a centrosomal bait protein of interest is fused to a promiscuous biotin ligase and expressed in the respective model cell line. The biotinylase then conjugates biotin residues to any lysine residue within a proximity of ~10 nm. Biotinylated proteins can then be identified and enriched by streptavidin conjugated to fluorophores (for microscopy) or beads (for affinity purification), respectively. Purified biotinylated target proteins are then identified by antibodies or mass spectrometry. Due to the extremely high affinity of the streptavidin-biotin interaction, BioID only rarely brings up false positive interactors. In centrosome research this robust method was applied in mammalian cells and *Dictyostelium* amoebae with great success [37,61,62,63,64]. In mammalian cells, these analyses also revealed a molecular relationship between so-called centriolar satellites, i.e., microscopically visible proteinacious granules of the pericentrosomal area. In this context, two centriolar satellite components, CCDC14 and KIAA0753, were identified as interactors of the centrosomal protein CEP63. As the latter interacts with the centriole duplication organizer CEP152 through the same protein domain this provides a mechanism how CCDC14 may negatively regulate centriole duplication [65]. Our current knowledge of the still somewhat mysterious centriolar satellites and their regulation is reported by Nielsen et al. in this issue of *Cells* [64]. This paper also emphasizes the importance of superresolution light microscopy techniques, which have been the major driving force for the elucidation of subcentrosomal protein topology within the last decade. These developments started in 1998 with one of the first successful applications of deconvolution fluorescence microscopy for the identification of pericentrin/γ-tubulin topology within the pericentrosomal matrix in the Doxsey lab [66]. Deconvolution is still the most economic method to overcome Abbe’s resolution limit in light microscopy of standard fluorescence specimens. In this computer-based method, a measured point spread function allows the re-calculation of diffracted fluorescence light to its origin. The power of this method is also described in this issue of *Cells* [37]. Further progress in the study of subcentrosomal topology came from hardware-based superresolution techniques, i.e., from structured illumination microscopy (SIM) [67,68,69], stimulated emission depletion microscopy (STED) [70], and direct stochastic optical reconstruction microscopy (dSTORM) [71]. In case of animal centrosomes these superresolution light microscopic analyses led to a model, in which pericentrin and CEP152 emanate from the mother centriole as radial spokes and act as a scaffold for toroid layers of the other PCM proteins [72]. Similarly, these methods, together with electron microscopy gave a detailed view on the arrangement of all SPB components within the major substructures of the SPB (i.e., inner, central, outer plaque, and half bridge; see [73] for a review).

## 6. Conclusions

This issue of *Cells* shows that the once mysterious organelle “centrosome” has disclosed many of its secrets, especially regarding its composition and microtubule organization. Still there are many open questions. How is the assembly of about a hundred different centrosomal components into a highly sophisticated topology regulated through various signaling pathways, how are centrioles/basal bodies involved in signaling at primary cilia, how are centrosomal proteins involved in the etiology of several devastating diseases and last not least, what is the evolutional relationship of centrosomes with nuclear pore complexes.
